# Cryptococcal Immune Reconstitution Inflammatory Syndrome: From Blood and Cerebrospinal Fluid Biomarkers to Treatment Approaches

**DOI:** 10.3390/life11020095

**Published:** 2021-01-27

**Authors:** Vânia Maria Sabadoto Brienze, Júlio César André, Elisabete Liso, Irina Vlasova-St. Louis

**Affiliations:** 1 Department of Neurological Sciences, Faculty of Medicine of São José do Rio Preto (FAMERP), 15090000 São Paulo, Brazil; eliliso@terra.com.br; 2 Department of Molecular Biology, Faculty of Medicine of São José do Rio Preto (FAMERP), 15090000 São Paulo, Brazil; julio.andre@famerp.br; 3 Department of Medicine, University of Minnesota, Minneapolis, MN 55455, USA

**Keywords:** immune reconstitution inflammatory syndrome (IRIS), AIDS/HIV, antiretroviral therapy (ART), cryptococcal meningitis (CM), blood biomarkers, cerebrospinal fluid biomarkers

## Abstract

Immune reconstitution inflammatory syndrome (IRIS) presents as an exaggerated immune reaction that occurs during dysregulated immune restoration in immunocompromised patients in late-stage human immunodeficiency virus (HIV) infection who have commenced antiretroviral treatments (ART). Virtually any opportunistic pathogen can provoke this type of immune restoration disorder. In this review, we focus on recent developments in the identification of risk factors for Cryptococcal IRIS and on advancements in our understanding of C-IRIS immunopathogenesis. We overview new findings in blood and cerebrospinal fluid which can potentially be useful in the prediction and diagnosis of cryptococcal meningitis IRIS (CM-IRIS). We assess current therapeutic regimens and novel treatment approaches to combat CM-IRIS. We discuss the utility of biomarkers for clinical monitoring and adjusting treatment modalities in acquired immunodeficiency syndrome (AIDS) patients co-infected with *Cryptococcus* who have initiated ART.

## 1. Introduction

*Cryptococcus* species are the most common cause of meningitis in adults and one of the leading causes of human immunodeficiency virus (HIV)-related mortality in the world, with a global incidence estimated at 223,100 cases per year [1,2]. In countries with limited resources, cryptococcosis drives up to 40% of all hospitalizations and deaths associated with the advanced stage of HIV infection [3,4,5]. Immune reconstitution inflammatory syndrome associated with cryptococcosis (C-IRIS) is a common complication that manifests after the initiation of antiretroviral therapy (ART) [6,7]. Cryptococcal IRIS presents as an exaggerated and deregulated proinflammatory immune reaction, which accompanies the reduction in peripheral blood HIV viral load and the initiation of CD4+ T cell recovery [7]. Approximately 25% of HIV and *Cryptococcus* co-infected patients develop cryptococcal meningitis IRIS (CM-IRIS) within the first four months of ART treatment, with an average mortality rate of around 20 +/− 10% [8].

There are two recognized forms of CM-IRIS. The first form is “unmasking” IRIS, in which the individual manifests an inflammatory response to *Cryptococcus spp.*, revealing previously undiagnosed cryptococcal meningitis (CM) in ART-naïve individuals after starting ART [7,9]. Unmasking CM-IRIS may include neurological symptoms driven by high intracranial pressure and inflammation, such as severe headaches, vomiting, visual impairment (diplopia, photophobia, blindness), hearing loss, seizures, ataxia, or aphasia [10,11,12]. Altered mental status, including personality and behavioral changes, confusion, hallucinations, and in rare cases, lethargy, are attributable to unmasking CM-IRIS. Unmasking IRIS is usually diagnosed within 2 to 6 weeks on antiretroviral treatment and it is the deadliest [13]. Thus, the diagnosis of cryptococcal infection is essential for the prevention of unmasking CM-IRIS. The second form is “paradoxical” IRIS, which occurs in the settings of induction antifungal therapy [6,14]. Paradoxical CM-IRIS became the most common due to significant improvements in the diagnosis of cryptococcal meningitis and the introduction of antifungal therapy regimens prior to ART commencement [6,15]. The paradoxical form also presents itself as neuro-cryptococcosis, with clinical symptoms of worsening neurological function impairments and altered mental status due to raised intracranial/cerebrospinal fluid (CSF) pressure. Paradoxical CM-IRIS can be clinically assessed according to Glasgow Coma Scores (GCS > 15) and via MRI imaging findings (e.g., multifocal or diffuse leptomeningeal and cortical enhancements) [14,16,17,18]. Paradoxical C-IRIS manifests, on average, 1 to 6 months after the initiation of ART, and it occurs in the background of initial clinical and microbiological response to antifungal treatment, as well as virologic response to ART [2,14,19]. As described below, periodic examination of cerebrospinal fluid was found to be helpful to diagnose and predict paradoxical CM-IRIS [20].

Pulmonary C-IRIS has been described primarily in *Cryptococcus neoformans* infection. Clinical manifestations include cough, dyspnea due to pneumonitis, pulmonary infiltrates, lymphadenopathy, cavitation, and nodular lesions [9,21,22]. Several components of the immune system such as T cells and macrophages, pro-inflammatory cytokines and chemokines are thought to be involved in the pathology of pulmonary C-IRIS, although they have not been systematically studied (reviewed in [23]).

In this review, we focus on recent advances in mitigating risk factors for C-IRIS, and the prognostic and diagnostic molecular biomarkers for better understanding CM-IRIS immunopathogenesis. New biomarkers may help to identify putative host-based targets to justify a clinical need for the improvement of laboratory monitoring and adjusting treatment modalities in acquired immunodeficiency syndrome (AIDS) patients co-infected with *Cryptococcus* who have initiated ART.

## 2. Conventional Risk Factors for CM-IRIS

The conventional risk factors of CM-IRIS can be divided into three categories.

### 2.1. Host-Related Risk Factors

Because the variety of opportunistic pathogens has been linked to ART-associated IRIS in people with acquired immunodeficiency syndrome (AIDS), host-related risk factors are considered most important and universal for several types of IRIS [24]. Improvements in immune status in patients who have been severely immunocompromised are often accompanied by disbalanced immune reconstitution, which represents a host risk factor for the development of IRIS. Patients, as such, have very low pre-ART CD4+ T cell count in the blood (<100 CD4+ cells/uL) [25,26] and high HIV viral load (>100,000 copies/mL of blood) [27]. They include low baseline antibody responses to *Cryptococcus spp.*, e.g., decreased levels of total plasma IgM and specific antifungal IgM (GXM-IgM or β-glucan-binding IgM) [28], and a lack of pro-inflammatory cytokines in the serum, or CSF, as described below. Genetic factors, such as single nucleotide polymorphism Interleukin 7 receptor subunit alpha (IL7RA), may affect predisposition in the development of IRIS [29]. Allelic polymorphisms (e.g., in CYP2C19 gene) may be considered as patient-specific risk factors affecting fungicidal drug activities, toxicity, and the level of inflammation (e.g., C-reactive protein or albumin levels) [30]. More recently, transcriptomic profiles have been assessed, and several molecular pathways were proposed (as potential baseline biomarkers), as described below [31]. Among readily available baseline biomarkers, low hemoglobin concentrations (<8.5 g/dL), high C-reactive protein levels (CRP > 32 mg/L) or D-dimers (>3.89 ug/mL) are predictive of IRIS events [8,15].

### 2.2. Pathogen-Related Risk Factors

The genomic differences in clinical isolates may underlay differential drug susceptibility and virulence of *Cryptococcal spps*., which play important roles in the severity of CM and CM-IRIS [32]. Genetic make-up allowed some *Cryptococcal spps*. advantaged metabolic fitness, as was tested in pre-clinical models [33,34,35]. Mutated species of HIV may also play roles in the pre-treatment drug resistance to non-nucleoside reverse transcriptase inhibitors ART, and in alterations of immune responses after ART initiation [36].

In recent years, there has been an international collaborative effort focused on the development of point-of-care assays in resource-limited laboratory settings [37,38]. The lateral flow assay (LFA, IMMY^®^ diagnostics, Norman, OK, USA) utilizes gold-conjugated monoclonal antibodies that target capsular polysaccharide glucuronoxylomannan (GXM), a primary cryptococcal antigen of four serotypes [39]. LFA is fast and able to quantify cryptococcal antigen (CrAg) titers with high sensitivity and specificity in CSF: 100% and 99.8%, respectively (reviewed in [40]). Cryptococcal antigenemia (CrAg+) in serum, CSF or other biofluids is often detected in asymptomatic CM patients who subsequently develop CM-IRIS [41,42,43,44]. Mortality also remains higher in CrAg+ immunocompromised patients after initiation of ART, and the levels of CD4+ T cells are inversely correlated with CrAg titers [45,46]. The FDA-approved BioFire FilmArray^®^ meningitis/encephalitis nested multiplex PCR panel (bioMérieux, Salt Lake City, UT, USA) has recently been introduced into routine clinical practice, and it can detect and differentiate DNA from *C. neoformans* and *C. gattii* (among other pathogens) [47].

With the development of highly sensitive and specific molecular tests, great advances have been made in the diagnostic procedures of CM caused by various Cryptococcal species. Isothermal molecular techniques, such as LAMP (Loop-mediated isothermal AMPlification), have also contributed to improving the diagnosis of fungal diseases. The LAMP technique is based on the principle of isothermal loop amplification to identify the species of *Cryptococci* from the CSF culture isolates targeting the internal transcribed spacer (ITS) region and CAP59 gene. The LAMP assay has high specificity for molecular genotypes VNI, VNII, and VNIII of *Cryptococcus neoformans*, and is able to differentiate from *C. gattii* and other fungal species [48]. LAMP does not require expensive laboratory instrumentation to perform, thus in the future it can be introduced as a point-of-care assay.

Culture remains a gold standard to assess live pathogens in the CSF or blood, by measuring colony-forming units (CFU/mL) growth on Sabouraud dextrose agar for 48 h at 30 °C. An important prognostic parameter, such as early fungicidal activity (EFA), can be calculated from recurrent cultures during induction regimens (described below). Microbiological clearance is measured as log_10_ clearance of *Cryptococcus* yeasts per mL of CSF and serves as an important predictor of increased mortality, including that from C-IRIS [49]. Cultured isolates can subsequently be serotyped by real-time PCR assay, and mating type can be determined by amplified fragment length polymorphism (AFLP) or PCR-restriction fragment length polymorphism (PCR-RFLP) [34,50]. Molecular typing revealed that genotypes, drug susceptibility, and the virulence of *Cryptococcus* species varied between different continents and in different countries [51]. However, recent studies found no correlation between antifungal drug susceptibility and hazards of death for therapeutic outcomes in the cohort of severely immunosuppressed AIDS patients [52].

### 2.3. Treatment-Related Risk Factors

Inadequacy of high-dose antifungal drug monotherapy or combination therapy is the number one treatment-related factor for IRIS, as mortality from CM is the highest during the first 6 months under routine protocols [53,54]. A combination of various antifungal regimens has been tested and compared in the procurement of the best antifungal activity in the shortest period of time [55,56]. However, a shorter duration between induction antifungal treatment and the initiation of ART may predispose patients to fatal C-IRIS events. The proposed explanation is an insignificant time for the achievement of microbiological clearance [54,57].

A combination of highly active antiretroviral drugs seems to have immunomodulatory effects, yet in some cases increases IRIS incidence, depending upon patient-specific factors [58]. The rapid decrease in HIV viral load on ART (>2.5 log reduction in IRIS patients, over 4 weeks, when compared to pre-ART viral load) has also been identified as a risk factor for activation of the host immune system and IRIS [27,59,60]. On the other hand, the interruption of ART or development of ART resistance slows down the immune reconstitution and prolongs chronic inflammation [61,62]. After ART initiation, confirmation of the virologic response is highly recommended for the diagnosis of treatment failure or suboptimum responses to combination ART, but it is not essential for the prediction of IRIS [63,64].

Thus, rapid cellular immune activation that drives the symptoms of CM-IRIS is predicated by a combination of several factors, such as the hosts’ immunological predisposition, the microbial antigen burden, and the effectiveness of the drugs (ART or antifungals).

## 3. Immunopathogenesis of C-IRIS

Human immunodeficiency virus infects and persists in CD4+ T cells, astrocytes, microglial cells, and less frequently, in cells of monocyte-macrophage lineage [65,66]. During the first few weeks on ART, the rate of HIV viral load decreases and T cell population stabilizes; however, the recovery initiates very slowly in severely immunodeficient individuals. The initiation of ART at the advanced stage of HIV infection (at CD4+ T cells ≤ 200/mm^3^) is associated with long-term immune and metabolic abnormalities (up to 3 years after ART initiation) [67]. In fact, the thymic output of naïve CD4+ T cells (detected as T cell receptor excision circles (TRECs)) exhibits an augmenting trend only after 12 months on ART in adults and young children [68,69]. Pre- and post-ART, CD4+, and CD8+ T cells express immune checkpoint molecules (e.g., PD-1, TIGIT, and LAG-3), which are associated with long-term HIV persistence and immune exhaustion [70]. During IRIS episodes, higher frequencies of PD-1+/CD4+ T cells expressing LAG-3, CTLA-4, and ICOS have also been detected when compared with patients without IRIS [71]. HIV-AIDS patients, who subsequently die from CM-IRIS or CM after ART initiation, also exhibit upregulation of immune checkpoint gene expression (PD-1, PDL-1) in peripheral blood at the time of ART (Vlasova-St. Louis, unpublished). The persistence of expression of immune checkpoint molecules and T cell immune exhaustion correlates with the increased size of the HIV reservoir, which seems to play an important role in the subsequent ART-driven activation of cytotoxic CD8+ T cells and antigen-presenting cells [72,73]. This may lead to unproductive and paradoxical immune reactions to a reservoir of antigens, secretion of pro-inflammatory mediators by activated monocytes and macrophages, and tissue damage [74]. Thus, the transition from the state of immune exhaustion and poor macrophage function to ART-related immune reconstitution is often accompanied by exaggerated innate inflammatory responses which lead to IRIS.

### 3.1. Blood Plasma and Serum Biomarkers in CM-IRIS

Blood transcriptomic profiles have been used to characterize changes in ART-induced gene expression between HIV-infected patients with CM who developed paradoxical CM-IRIS and those who did not [31,75]. The analysis of these profiles showed that, before starting ART, patients who eventually developed CM-IRIS exhibit a significant decrease in the expression of transcripts encoding type I interferons (IFN I) and antiviral defense proteins, particularly those that lead to the downregulation of viral replication, while antimicrobial defense genes are upregulated [31]. Earlier observation of high plasma levels of soluble CD40L, which is known to suppress IFN-α production, may provide an explanation for INF type I deficiency in C-IRIS patients [76]. Prior to ART, patients with CM-IRIS also showed a reduction in interferon-gamma (IFN-γ) gene expression and IFN-γ secretion by the stimulated mononuclear cells in response to cryptococcal mannoproteins [77]. Interestingly, the most recent study led by Vlasova-St. Louis, which specifically focused on fatal CM-IRIS cases, identified that baseline IFN-γ expression has been elevated in patients who subsequently died from CM-IRIS (the manuscript is in print, BMC Medical Genomics 2021). Thus, the expression of components of antiviral defense pathways, such as type I/II interferons and IFN-induced genes, could be used as a predictive biomarker for fatal and non-fatal CM-IRIS ([31] and unpublished).

Blood granulocyte activation status, particularly neutrophils’, has been correlated with high mortality, including that from CM-IRIS, perhaps reflecting systemic oxidative stress generated by these short-lived effector cells of the innate immune system [78]. Transcriptomic biomarkers for activated granulocytes (e.g., oxidases, arginase, integrins, etc.) have been found to precede CM-IRIS events, which are accompanied by markers of tissue destruction (e.g., matrix metalloproteinases) [31]. Markers of monocyte activation (e.g., soluble CD14) in plasma correlate positively with levels of IL-6, C-reactive protein, serum amyloid A and D-dimer, and are associated with higher mortality rates [79]. Research studies have also shown that a larger percentage of activated human CD14+CD16++ monocytes produces high amounts of TNF-α and IL-6, before and after IFN-γ co-stimulation ex vivo [80]. Higher frequencies of activated CD14+CD86+ or CD14+HLA-DR blood monocytes have also been observed in C-IRIS patients who failed to clear *Cryptococci* from CSF pre-ART [81]. In addition, inappropriately polarized and activated macrophages may persistently harbor a residual viral replication during ART due to enhanced expression of efflux transporters [82]. Altogether, this may represent a source of pro-inflammatory cytokines such as IL-6, which can be measured in abundance in patients’ blood during CM-IRIS events [83].

Excessive expression of chemokines and integrins in peripheral blood precedes CM-IRIS and can be assessed by monitoring patients during pathological immune reconstitution [31]. Plasma levels of cytokines, such as IL-6, IL-18, TNF-α, IL-5, IL-7, IL-17, GM-CSF, CCL11, and CXCL10, are increased in patients who develop CM-IRIS [84,85,86]. These cytokines represent mediators of a systemic immune response and local inflammatory events that occur in the central nervous system (CNS) [86]. To mitigate the cytokine storm during CM-IRIS, several antibody-based biologics have been studied, as described below. Additionally, CXCR7 has recently been proposed as a potential therapeutic target receptor on CD14+CD16+ monocytes that may limit neuroinflammation by controlling the activated patrolling monocyte entry to CNS [87].

The baseline (pre-ART) absolute number of B cells in the peripheral blood is not different between patients who subsequently developed CM-IRIS and those who did not. However, low plasma levels of IgM antibodies secreted toward the cryptococcal polysaccharide antigens, glucuronoxylomannan (GXM), laminarin, and pustulan represent poor B cell function and may put patients at high risk of CM-IRIS [28]. Poor antibody response to cryptococcal antigens indicates the important role of antibody-mediated cryptococcal antigen clearance. A longitudinal study using blood leucocytes from patients with HIV and CM (with and without IRIS) showed disproportionate cellular expansion of CD66+CD16+/− neutrophils and deactivated monocyte subpopulations, but no increases in T-helper cell type 1 populations after antifungal treatment, which conferred the inability to clear *Cryptococcal* antigens [84,88]. Peripheral blood from patients who had fatal outcomes revealed low GXM- and LPS-driven monocyte responses (CD16+/−HLA-DR^low^) and reduced TNF-α, but increased IL-6, IL-10, and CXCL10 production [88]. In the setting of severe lymphopenia, the decreased phagocytosis, antigen-presentation, and failure to properly clear antigenic load result in sequential exaggerated responses during the early phase of CD4+ T-cell reconstitution [89].

IL-7/IL-7R interaction plays a homeostatic role in T cell survival and repopulation during the initial phase of immune recovery, and abnormal IL-7 plasma levels are strongly associated with CM-IRIS [81]. Dysbalanced cytokine milieu may inappropriately differentiate T helper 0 cells into Th1 and Th2 types, which, in turn, impairs the adaptive immune response to *Cryptococcus spp.* [83,90]. Recent genetic studies revealed that the T allele homozygosity at rs6897932 of the L-7R gene (encodes CD127) links to a faster CD4+ T cell recovery after ART initiation, as opposed to CC genotypes in HIV-infected individuals [29,91]. Future genotyping studies of cytokine and cytokine receptor genes may reveal the link between the single nucleotide polymorphism in AIDS patients and predisposition to immune reconstitution disorders, such as IRIS [92,93,94,95].

There are limited studies that assessed blood biomarkers before, during, and after CM-IRIS events. The differences between molecular phenotypes of CM-IRIS events have been identified via assessment of the kinetics of transcription of immune genes. Two molecular subtypes of CM-IRIS events have been identified: early (occurred within two months on ART) and late CM-IRIS (occurred after 3 months on ART). Early CM-IRIS has been associated with an upregulation of the innate immune pathways: toll-like receptors (TLR) and inflammasome components (AIM2, CASP5, NLR receptors), and may in the future be classified as biomarkers of early CM-IRIS events. The late CM-IRIS showed a gene expression signature of an adaptive immune activation, in addition to innate immune biomarkers [31]. The findings of this study suggested that in late CM-IRIS the cells of adaptive immunity were not able to properly communicate with innate immune cells due to delayed maturation and functional recovery. Taken together, these data demonstrate strong involvement of the innate and adaptive immune axes in cryptococcal IRIS (Table 1).

### 3.2. Cerebrospinal Fluid Biomarkers of CM-IRIS

CNS injury is a hallmark of CM and CM-IRIS. Accumulating evidence suggests the involvement of microglia inflammation and the recruitment of naïve T cells in the brain during immune restoration, causing CNS pathology [96]. During a CM-IRIS episode, the phenotype of mononuclear cells in cerebrospinal fluid (CSF) demonstrates a CNS-compartmentalized shift from classic (CD14++CD16−) to an intermediate/pro-inflammatory phenotype (CD14++CD16+) [97]. However, immunosuppressive ligand PD-L1 expression is also high in both the CSF monocytes and CD56^dim^ and CD56^bright^ NK cells subsets, reflecting CNS immunopathology behind C-IRIS [97].

Paradoxically, weak cellular inflammatory responses in the CSF at the time of ART initiation have been observed to be predictive of CM-IRIS (Table 2) [98]. In patients with HIV and CM, low CSF IFN-γ, IL-5, IL-6, and G-CSF concentrations are associated with higher fungal load, the presence of double-negative CD4-/CD8-T cells, and increased mortality [99]. In severe cases of CM, the infected monocytes/macrophages have expressed alternative activation cluster differentiation markers (e.g., CD206, CD163, CD200) and have been unable to eradicate *Cryptococcus spp.* from CSF, spreading the pathogen to the central nervous system [99,100]. Examination of the associations between immune phenotype in the CSF and clinical outcomes, particularly death and CM-IRIS, revealed two divergent routes. The first is the protective immune route that is driven by high pre-ART CSF levels of IL-6, IL-8, IL-10, IL-17, TNF-α, and IFN-γ [101]. This route aims to decrease the fungal burden and improve microbial clearance, but has shown to be deficient in CM-IRIS patients. The second route is represented by high levels of CCL2 (MCP-1), CCL3 (MIP-1α), and GM-CSF that are secreted by CNS-resident monocytes and are associated with the subsequent development of CM-IRIS [101]. These cytokines/chemokines can increase the traffic of CD4+ T cells and myeloid cells to the CNS and CSF. It has been demonstrated that after the initiation of effective antifungal therapy, there has been an enrichment for CD8+ T cells that co-express CXCR3 and CCR5 receptors. Additionally, the increase in CCL2/CXCL10 and CCL3/CXCL10 ratios has been observed in the CSF of CM patients with neurological deteriorations who subsequently developed CM-IRIS on ART [102]. The trafficking of CD8+T cells and chemokines into the CSF is probably expedited through the damaged blood-brain barrier caused by ongoing chronic inflammation [102]. Soluble macrophage-specific activation biomarkers (e.g., sCD163, sCD14, CCL3) are abundant in the CSF of patients who are at a higher risk of mortality from CM-IRIS, which also suggests macrophage/microglial involvement in recruiting cytotoxic cells to CNS during CM-IRIS pathogenesis [103].

In addition to opportunistic infections, patients with advanced HIV infections suffer from HIV associated neurocognitive disorders (HAND). Cryptococcal meningitis exacerbates neurocognitive impairment in these patients. HAND patients already exhibit a high percentage of TNF- and IFNγ-expressing T cells, increased levels of sCD163 and sCD14, and have low degranulation capacity of CD8+CD107+ T cells. Thus, the additional cryptococcal burden may exaggerate CSF immune responses, the influx and retention of activated immune cells and proinflammatory mediators, which contribute to high intracranial pressure during CM-IRIS pathogenesis [104].

Based on the assessment of 21 biomarkers, it has been suggested that patrolling monocytes and CNS residing monocytes have distinct chemokine expression and cytokine production profiles [105]. The macrophages can also be present at different polarization states in peripheral blood and CNS. Although not readily available in human subjects, CNS-resident macrophages may be the most relevant source of pro-inflammatory biomarkers and the main drivers of immunopathology [106]. Considering that the data on the pathogenesis of CM-IRIS are derived from analyses of few immunological parameters, further comprehensive studies are needed to understand whether the baseline and the kinetics of inflammatory response before and during C-IRIS, in different body compartments, may help us to identify biomarker panels that may be useful in clinical settings.

## 4. Treatment Advances for the Management of Cryptococcal Infection and C-IRIS

With respect to therapies, there are still no effective treatments for C-IRIS; however, targeting the risk factors described in 2.2 may decrease IRIS incidence and severity. The CM treatment regimens are composed of three pharmaceuticals: amphotericin B deoxycholate (AmB) or liposomal AmB (L-Amb), flucytosine (5-FC), and fluconazole (FLU) (Table 3). The treatment of CM is divided into three phases: induction, consolidation, and maintenance (reviewed in [107]).

The induction phase aims to drastically decrease the fungal burden in the patient’s cerebrospinal fluid in the first 2 weeks and is fundamental for survival. AmB has high toxicity at standard doses of 1 mg/kg/day, such as hepatic and renal toxicity, anemia, electrolytic abnormalities, or reactions at the site of infusion. For this reason, a recent formulation of liposomal amphotericin B (L-AMB) has been recommended as it is less toxic at a single dose (10 mg/kg), has a longer half-life, and can more effectively penetrate the brain tissues [108,109,110]. Presently, the combination AmB/5-FC provides the most effective fungicidal activity and cryptococcal clearance [112]. A novel formulation of oral AmB is currently being tested for safety and tolerability in the EnACT Trial [111].

Voriconazole (VCZ) is an antifungal agent that is used to treat invasive fungal infections, such as cryptococcosis, aspergillosis, and candidiasis. A study conducted in South Africa assessed its efficacy in treating CM and showed no statistically significant difference between the use of AmB/FLU or AmB/voriconazole and the standard AmB/5-FC therapy [113,114]. VCZ has good bioavailability, but owing to a higher cost, scarcity of studies on CNS penetration, and altered pharmacokinetics in the context of inflammation, its use is limited [115]. Although other triazoles such as itraconazole, posaconazole, and prodrug isavuconazole [116,117] exhibit anticryptococcal activity, they are only used as a second-line agent or in combination with AmB due to drug–drug interactions and toxicity (reviewed in [118]).

In the consolidation phase (2–6 weeks of induction therapy), doses of antifungal agents are decreased, and antiretroviral therapy is initiated. The initiation of the consolidation phase and ART must be considered carefully [123]. The introduction of ART is recommended 4–6 weeks after starting induction antifungal therapy to improve survival rates and achieve sustained clinical responses [124]. A pragmatic approach to the management of patients with HIV-associated cryptococcal meningitis has been outlined in a recent study [125]. Recently, a new tetrazole compound, VT-1129 (Viamet Pharmaceuticals Inc.), has been shown to exhibit potent in vitro activity against *Cryptococcus spp*. [119]. VT-1129 is highly selective for fungal CYP51, has minimal effect on human cytochrome P450 enzyme metabolism, and may potentially be used as a preemptive or consolidation therapy for fluconazole-resistant cryptococcal meningitis [120].

The maintenance phase is introduced to maintain the sterility of CSF culture and to prevent a relapse of cryptococcal disease. The fluconazole maintenance therapy (200 mg/day) is extremely important; however, this phase is the most vulnerable to non-compliance and loss to follow-up [121,122].

There are three main approaches to combat C-IRIS: supportive care aiming to reduce host-related risk factors (described in Section 2.1), symptomatic treatment of high intracranial pressure in cases of CM-IRIS, and anti-inflammatory or immunomodulatory approaches to diminish inflammation.

The symptoms of paradoxical CM-IRIS are accompanied by abnormal radiographic imaging findings (described in Section 1) and high intracranial pressure (>250 mm H2O) [14]. Thus, as a symptomatic treatment, therapeutic lumbar punctures and, in some cases, shunting are recommended for those suspected of CM-IRIS [126,127]. Nonsteroidal anti-inflammatory drugs (NSAIDs) are widely used in the cases of mild and self-contained forms of CM-IRIS. In cases of severe symptoms of CM-IRIS, the administration of corticosteroids (particularly dexamethasone) has been found to be beneficial as it decreases inflammation, although it has also been shown to be associated with higher mortality [106,128,129]. In cases of pulmonary cryptococcal IRIS, corticosteroid treatment may be considered in the event of the development of respiratory distress, but the antifungal regimens should be continued [119,120]. The immunosuppressive drug hydroxychloroquine reduces lipopolysaccharide/TLR-mediated immune signaling, which may be important for CM-IRIS prevention, as early CM-IRIS is solely driven by innate immune activation pathways, especially in immunological non-responders (patients with CD4+ T cell increase of <5% in the last 12 months on ART) [130] (Table 4).

Other immunomodulatory agents (such as thalidomide or adalimumab) have been tested in severe cases of CM-IRIS. Several reports documented neurological improvement after the use of thalidomide and adalimumab—human monoclonal antibodies that bind to TNFα and block its anti-inflammatory activity [131,132]. Another biologic, a recombinant IFNγ, has been shown to expedite CSF fungal clearance by increasing Th1 cell responses and depolarizing macrophages, although it has failed to exhibit evident benefits to patient survival [133,134]. In all cases of C-IRIS, ART should be continued unless there is a risk of fatal outcomes [135,136] (Table 4).

## 5. Conclusions

Herein, we discussed immune reconstitution inflammatory syndrome in patients co-infected with HIV and cryptococcosis, with particular attention to clinical presentation, risk factors, immunopathology, and treatment. Mortality from CM-IRIS can be reduced significantly if patients initiate antiretroviral therapy during the phase of moderate immunosuppression and before significant CD4+ T cell count loss [137,138]. The morbidity and costs associated with IRIS in people living with HIV have continued to decline in the USA since 2012 (<8%), with no fatality [3]. Although much research is being done on the mechanisms that modulate the immunopathogenesis of CM-IRIS, no biomarkers for this condition have been validated to produce a sufficient level of evidence to enter clinical practice. The recently proposed EQUAL Cryptococcus score, as a guideline for the optimal management of cryptococcosis, includes intervention steps in CM-IRIS cases [139]. Future frontiers for more effective therapy appear to be to: (1) improve the time to HIV diagnosis; and (2) identify and manage associated immune-inflammatory conditions. The development of effective antifungal medications, companion immunomodulatory therapies, and the improvement of patients’ healthcare in resource-limited countries should be a priority for the next few decades.

## Figures and Tables

**Table 1:** Blood biomarkers in human immunodeficiency virus (HIV)-infected patients with cryptococcal meningitis (CM) who developed cryptococcal meningitis immune reconstitution inflammatory syndrome (CM-IRIS).

Phase	Biological Activity	Biomarkers	Reference
Prior to ART initiation	Granulocyte activation, systemic oxidative stress	↑ oxidases, arginases, integrins, matrix metalloproteinases (MMP), etc.	Musubire et al., 2018 [78] Vlasova-St. Louis et al., 2018 [31]
Prior to ART initiation	Transcriptomic profiles of peripheral mononuclear cells	↓ type I interferons (IFN I), antiviral defense gene expression	Vlasova-St. Louis et al., 2018 [31]
	In vitro stimulated mononuclear cells	↓ interferon-gamma (IFN-γ)	Chang et al., 2013 [77]
	Activated monocyte status Activated CD14+CD16++ monocyte	↑ IL-6, C-reactive protein, serum amyloid A and D-dimer, sCD14, ↑ TNF-α and IL-6	Sandler et al., 2011 [79] Meya et al., 2017 [80]
	Increased levels of blood cytokines and chemokines	IL-6, IL-18, TNF-α, IL-5, IL-7, IL-17, GM-CSF, CCL11, and CXCL10	Meya et al., 2019 [84] Rateni et al., 2018 [85] Akilimali et al., 2017 [86]
	Poor B cell function upon stimulation with	↓ IgM antibodies secretion in response to Cryptococcal antigens	Yoon et al., 2019 [28]
At the time of CM-IRIS events	Transcriptomic profiles of peripheral mononuclear cells	Early CM-IRIS: ↑ inflammasome components: IL1B, Casp1, AIM2 NAIP, NLRP3, etc. Late CM-IRIS: ↑ IFNG, IL27, KLRB1, CD3, CD247, CD8, IL2RB	Vlasova-St. Louis et al., 2018 [31]

Abbreviations: ↑ increase; ↓ decrease; CM-IRIS—cryptococcal meningitis IRIS; ART—antiretroviral therapy; IFN—interferon; IL—interleukin; TNF—tumor necrosis factor; GM-CSF—granulocyte-macrophage colony-stimulating factor; CNS—central nervous system; sCD14—soluble CD14.

**Table 2:** Cerebrospinal fluid biomarkers in HIV-infected patients with CM who developed CM-IRIS.

Phase	Biological Activity	Biomarkers	Reference
Prior to ART initiation	Pluricellular CSF, low immune response	↓ WBC; ↓ TNF-α, CCL11, ↓ IFN-γ, VEGF, G-CSF, CCL2; ↓ CD4+ CD4−CD8− T cells, NK cells; ↓ IL-5, IL-6, G-CSF, IFN-γ; ↓ CD4^+^; ↓ IL-6, IL-8, IL-10, IL-17, TNF-α, and IFN-γ	Boulware et al., 2010 [98] Scriven et al., 2017 [100] Jarvis et al., 2015 [101]
	Activation of CD8+ T cells	↑ CCL2/CXCL10, ↑ CCL3/CXCL10, ↑ CXCR3 CD8+, ↑ CCR5 CD8+	Chang et al., 2013 [102]
	Activation of monocytes	↑ CCL2 (MCP-1), ↑ CCL3 (MIP-1α), ↑ GM-CSF, CCL5	Jarvis et al., 2015 [101]
	Macrophage/microglia activation	↑ sCD163, sCD14, CCL3	Scriven et al., 2015 [103]
At the time of CM-IRIS events	Activation of mononuclear cells in the background of immune exhaustion	↑ PD-L1 ↑ CD14++CD16−, ↑ CD14++ CD16+; ↑ HLA-DR+CD4+ ↑ CD56^bright^ NK cells	Meya et al., 2015 [97]

Abbreviations: ↓ decrease; ↑ increase; WBC, white blood cells; sCD163, sCD14, soluble CD163 and CD14.

**Table 3:** Treatment advances for the management of cryptococcal infection with antifungal drugs.

Drug	Findings	Reference
Amphotericin B deoxycholate (AmB), 1 mg/Kg/day	Hepatic and renal toxicity Side effects: Anemia Electrolytic abnormalities Reactions at the site of infusion	Lawrence et al., 2018 [108] Molloy et al., 2018 [109] Molefi et al., 2015 [110]
Liposomal AmB (L-AmB) 10 mg/Kg	Less toxic Single dose administration Longer half-life More effectively penetrates the brain tissues	Lawrence et al., 2018 [108] Molloy et al., 2018 [109] Molefi et al., 2015 [110]
Encochleated amphotericin B deoxycholate (cAMB) 1–2 g per day	In test-EnACT Trial	Skipper et al., 2020 [111]
Flucytosine (5-FC) 50–150 mg/kg/day	Provides most effective fungicidal activity when combined with AmB	Concha-Velasco et al., 2017 [112]
Voriconazole (VCZ) 400 mg/day	Good bioavailability Higher cost No statistically significant differences between VCZ and AMB/FLU or AmB/5-FC	Loyse et al., 2012 [113] Li et al., 2016 [114] Zeng et al., 2020 [115]
Posaconazole Isavuconazole Itraconazole	Used as a second-line agent in combination with AmB Exhibit drug–drug interactions and toxicity	Wong et al., 2020 [116] Jørgensen et al., 2019 [117] Houšť et al., 2020 [118]
VT-1129	Highly selective for fungal CYP51 Minimal effect on human cytochrome P450	Lockhart et al., 2016 [119] Nielsen et al., 2017 [120]
Fluconazole (FLU) 200 mg/day	Maintenance phase of CM treatment	Quan et al., 2019 [121] Bongomin et al., 2018 [122]

Abbreviation: CM—cryptococcal meningitis.

**Table 4:** Treatment advances for the management of CM-IRIS with anti-inflammatory agents and biologics.

Drug/Treatment	Findings	Reference
Therapeutic lumbar punctures/shunting	Recommended for high intracranial pressure	Govender et al., 2019 [126] Cherian et al., 2016 [127]
Corticosteroids (dexamethasone, prednisone, prednisolone) 0.3–1 mg/kg/day	Decreases inflammation, but associated with higher mortality Reduces TLR-mediated immune activation	Beardsley et al., 2019 [106] Day et al., 2014 [128] Beardsley et al., 2016 [129]
Hydroxychloroquine 400 mg/day	Neurological improvement in severe C-IRIS cases	Piconi et al., 2011 [130]
Thalidomide/Adalimumab 100 mg per day	Bind to TNFα and block its anti-inflammatory activity Expedites fungal clearance	Brunel et al., 2012 [131] Gaube et al., 2016 [132]
Recombinant IFNγ (Immukin, Boehringer Ingelheim) 100–200 μg s.c.	Increases Th1 cell responses Depolarizes macrophages No evident benefit to patient survival	Jarvis et al., 2012 [133] Gamaletsou et al., 2012 [134]

## Data Availability

Not applicable.

## Abbreviations

AIDS	acquired immunodeficiency syndrome
AmB	amphotericin B
ART	antiretroviral therapy
CM IRIS	cryptococcal meningitis IRIS
CNS	central nervous system
CrAg	Cryptococcal antigen
CSF	cerebrospinal fluid
FC	flucytosine
FLU	fluconazole
G-CSF	granulocyte colony-stimulating factor
GM-CSF	granulocyte-macrophage colony-stimulating factor
GXM	glucuronoxylomannan
HIV	human immunodeficiency virus
ICOS	inducible T cell costimulatory
IFN	interferon
IFN-γ	interferon gamma
IL	interleukin
IRIS	immune reconstitution inflammatory syndrome
LAG3	Lymphocyte Activating 3
LAmB	liposomal amphotericin B
LAMP	Loop mediated isothermal AMPlification
LFA	lateral flow assay
LPS	lipopolysaccharide
NSAIDs	nonsteroidal anti-inflammatory drugs
PD	programmed death
PD-L1	programmed death ligand 1
Th	T helper cells
TIGIT	T Cell Immunoreceptor With Ig And ITIM Domains
TLR	toll-like receptor
TNF	tumor necrosis factor
VCZ	voriconazole
